# Rainfall, groundwater, and surface water isotope data from extreme tropical cyclones (2016-2019) within the Caribbean Sea and Atlantic Ocean basins

**DOI:** 10.1016/j.dib.2020.105633

**Published:** 2020-04-30

**Authors:** Kristen Welsh, Ricardo Sánchez-Murillo

**Affiliations:** a Pure and Applied Sciences, University of The Bahamas, N-4912, Nassau, Bahamas; b Stable Isotopes Research Group and Water Resources Management Laboratory, Universidad Nacional, Heredia 86-3000, Costa Rica

**Keywords:** Tropical cyclones, Extreme rainfall, Stable isotopes, Caribbean Sea and Atlantic Ocean basins, Groundwater recharge, Climatic forcing

## Abstract

Under a changing climate, projections estimate that over the next thirty years, extreme Tropical Cyclones (TCs) will increase in frequency, with two to three times more Category 4 and 5 hurricanes in the Atlantic basin between 20°N and 40°N. In recent years, the Caribbean Sea and Atlantic Ocean basins have experienced several extreme TCs, resulting in extensive human, ecological, and economic damage [1], [2], [3]. To improve understanding of TCs and their potential impacts in the face of climate change, physically based understanding of past climate and modern TC dynamics is necessary. Despite the well-known Atlantic hurricane season, surface observations of the isotopic evolution of TC's moisture and the propagation of isotopically distinct pulses across surface and subsurface water reservoirs are lacking. In this data article, we provide novel high frequency sampling of surface rainfall isotope compositions (δ^18^O, δ^2^H, and *d*-excess in ‰) for Hurricanes Otto (Costa Rica, 2016), Nate (Costa Rica, 2017), Irma (Cuba and The Bahamas, 2017), Maria (Cuba and The Bahamas, 2017), and Dorian (The Bahamas, 2019). These five TCs were characterized by unprecedented impacts during continental and maritime landfalls and passages. In total, 161 surface rainfall samples were collected in passive devices [4] with event-based and daily frequencies, resulting in the first surface isotopic tempestology anatomy across the Caribbean Sea and Atlantic Ocean basins to date. Derived rainfall from TCs often results in large input amounts of isotopically distinct water over an area from few hours to several days, and therefore this unique isotope composition is propagated through surface and shallow subsurface reservoirs. Our data also include spring (N=338) and surface water (N=334) isotope compositions following the impact of Hurricane Otto and Tropical Storm Nate in central Costa Rica. As this region is well-known for its diverse rainfall dynamics and as a climate change ‘hot spot’ [5], [6], [7], our data provide an opportunity to improve and complement modern and past climate interpretations often derived from satellite products and calcite-δ^18^O paleoclimatic archives in light of climatic forcing, TC rainfall amounts and recharge rates, and the hypothesized climatic-induced decline of past Mesoamerican civilizations.

**Specifications Table**

**Table:** 

**Subject**	Analytical Chemistry, Atmospheric Science, Environmental Chemistry, Hydrology, Global and Planetary Change
**Specific subject area**	Stable isotope tempestology and extreme tropical cyclones
**Type of data**	Graphs
**How data were acquired**	Laser spectroscopy for water stable isotope analysis with a DLT-100 and IWA-45EP water analyzers (Los Gatos Research, Inc., California, USA) as well as a L2120-i water analyzer (Picarro Inc., California, USA).
**Data format**	Raw Analyzed
**Parameters for data collection**	Both rainfall collection in passive funnel devices [4] and manual/automated spring and surface water sampling ensured negligible secondary kinetic fractionation. Rainfall samples were collected in central Costa Rica, Cuba (Cienfuegos), and The Bahamas (Nassau) with discrete time intervals (i.e., event-based, 6-hourly and daily) prior to, during, and after TCs continental and maritime landfalls or passages. Spring (Pacific slope of central Costa Rica) and surface water (Caribbean slope of central Costa Rica) samples were collected on a daily/weekly (regular sampling frequency) and a 6-hourly basis during the direct and indirect influence of Hurricane Otto and Tropical Storm Nate in Costa Rica, respectively. Grab samples were collected on a weekly basis, using an automated peristaltic sampler AS950.
**Description of data collection**	Field work was conducted to collect event-based or daily rainfall samples with a passive funnel device [4] at each site, as well as manual and automated spring and surface water samples in central Costa Rica. All samples were sealed and refrigerated at 5°C in 30mL HDPE bottles until analysis at the Stable Isotopes Research Group laboratory, Universidad Nacional (Heredia, Costa Rica) and the Stable Isotope Laboratory at the Northern Rivers Institute, University of Aberdeen (Aberdeen, Scotland). The latter was used only for Hurricane Otto's samples.
**Data source location**	Institution: Universidad Nacional City/Town/Region: Heredia Country: Costa Rica Latitude and longitude (and GPS coordinates) for collected samples/data: 10.094533, -84.058700 Elevation: 1,949 m asl. Institution: Centro de Estudios Ambientales de Cienfuegos City/Town/Region: Cienfuegos
	Country: Cuba Latitude and longitude (and GPS coordinates) for collected samples/data: 22.065699, -80.483245 Elevation: 35 m asl. Institution: University of The Bahamas City/Town/Region: Nassau, New Providence Country: The Bahamas Latitude and longitude (and GPS coordinates) for collected samples/data: 25.073861, -77.413820 Elevation: 11 m asl.
**Data accessibility**	Repository name: Mendeley Data Data identification number: 10.17632/vwgffx5cv3.1 Direct URL to data: https://data.mendeley.com/datasets/vwgffx5cv3/1
**Related research article**	Data from TCs Otto, Nate, Maria, and Irma should be cited as: Sánchez-Murillo, R., Durán-Quesada, A.M., Esquivel-Hernández, G. *et al.,* Deciphering key processes controlling rainfall isotopic variability during extreme tropical cyclones, Nat. Commun. 10**,** (2019) 4321. https://doi.org/10.1038/s41467-019-12062-3[8]. Data of stable isotope compositions of the Pacific slope spring in Central Costa Rica should be cited as: Salas-Navarro, J., Sánchez-Murillo, R., Esquivel-Hernández, G., and Corrales-Salazar, J.L., Hydrogeological responses in tropical mountainous springs, Isotopes in Environmental and Health Studies, 55:1 (2019), 25-40, DOI: 10.1080/10256016.2018.1546701 [9] Data of stable isotope compositions of a Caribbean slope stream in Central Costa Rica should be cited as: Sánchez‐Murillo, R., Romero‐Esquivel, L. G., Jiménez‐Antillón, J., Salas‐Navarro, J., Corrales‐Salazar, L., Álvarez‐Carvajal, J., et al., DOC transport and export in a dynamic tropical catchment. Journal of Geophysical Research: Biogeosciences, 124 (2019), 1665– 1679. https://doi.org/10.1029/2018JG004897[10]

**Value of the Data**

• Our data provide the first high frequency surface stable isotope compositions of multiple tropical cyclones within the Caribbean Sea and Atlantic Ocean basins.
• Our data can be used in concert with other available satellite or radar products to assist with the understanding of key processes governing tropical storm genesis, development, and dissipation in a changing climate.
• Caribbean Sea and Atlantic Ocean modern and high frequency rainfall isotope data are available to be compared against other tropical cyclones in southern Asia and the western Pacific Ocean warm pool.
• Groundwater recharge from extreme tropical cyclones rainfall is emerging as a highly important component of the hydrological cycle in a changing climate. Therefore, our data offer a new venue to evaluate the impact of isotopically distinct pulses in shallow and deep groundwater reservoirs.
• Hurricanes Otto, Nate, Irma, Maria, and Dorian were among of the strongest tropical cyclones in recorded history to affect Costa Rica, Cuba, Puerto Rico, and The Bahamas, leading to the devastation of several locations, including the islands of Abaco and Grand Bahama during Hurricane Dorian and the northern lowlands of Costa Rica during Hurricane Otto. Our dataset represents a unique water fingerprint of these extreme events.
• More research into the isotopic evolution of tropical cyclones can better inform scientists about the hydrologic processes that influence these types of storms, which is particularly important as hurricanes are expected to increase in strength under a warming climate.

## 1 Data Description

**Figure 1:**
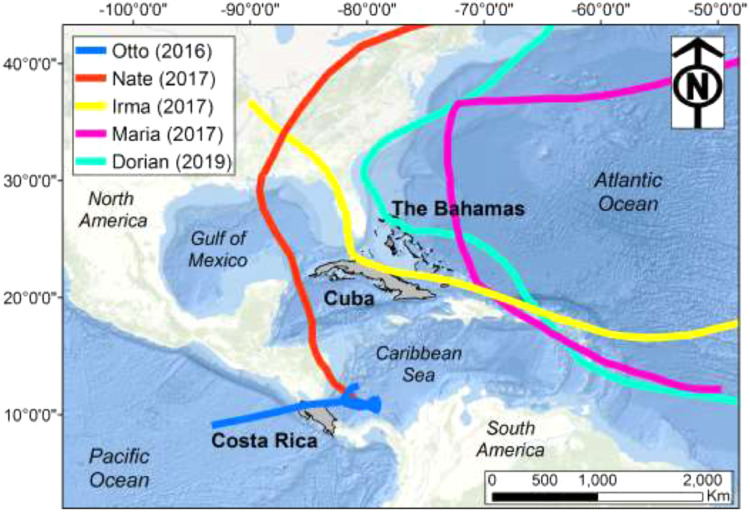
Countries where data were collected include central Costa Rica, Cuba (Cienfuegos), and The Bahamas (Nassau) within the Caribbean Sea and Atlantic Ocean basins. Each country polygon is grey-shaded. Bold lines represent the best track for all storm trajectories [16]. Landfalls represented by trajectories covering a portion of a country polygon are Otto (Costa Rica, 2016), Irma (Cuba, 2017), and Dorian (The Bahamas, 2019).

Figure 1 shows a map of countries where data were collected for this study: central Costa Rica, Cuba (Cienfuegos), and The Bahamas (Nassau) within the Caribbean Sea and Atlantic Ocean basins. Storm trajectories for Otto (2016) [11], Nate (2017) [12], Irma (2017) [13], Maria (2017) [14], and Dorian (2019) [15] were constructed from a combined dataset of the National Hurricane Center (NHC) archive and the Hurricane Databases (also known as HURDAT) (https://www.aoml.noaa.gov/hrd/hurdat/Data_Storm.html) [see 16, for more details about this database].

**Figure 2:**
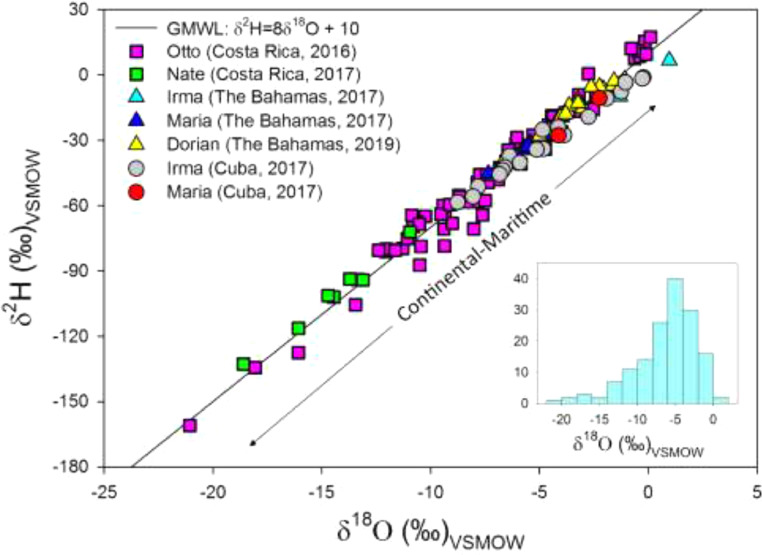
Dual isotope diagram showing the evolution of TCs (color and symbol coded) within the Caribbean Sea and Atlantic Ocean basins. Enriched compositions were identified in maritime landfalls and passages, whereas TC-continental land interaction was represented by more depleted compositions. The Global Meteoric Water Line (GMWL; black line) is included as a reference [17]. The inset shows a right-skewed histogram for all δ^18^O compositions.

Figure 2 presents a dual isotope diagram including the isotopic evolution of five major tropical cyclones: Otto (2016), Irma (2017), Maria (2017), Nate (2017), and Dorian (2019) relative to the Global Meteoric Water Line (GMWL) [17]. The inset in Figure 2 represents a right-skewed histogram for all δ^18^O compositions. Maritime TC passages include Hurricanes Nate and Maria, and continental landfalls include Hurricanes Otto, Irma, and Dorian.

**Figure 3:**
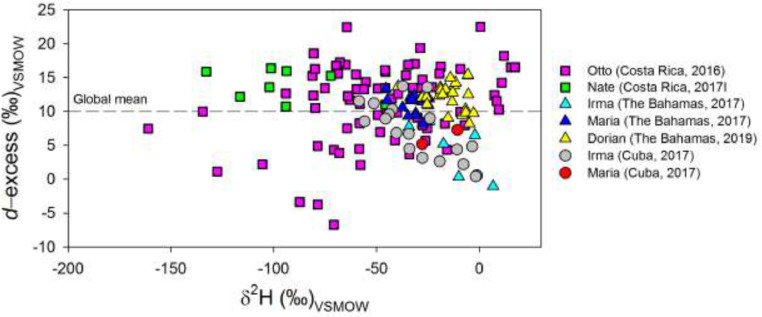
Dual isotope diagram showing *d*-excess variability (‰) for all sampled TCs (color and symbol coded) within the Caribbean Sea and Atlantic Ocean basins. The global mean of *d*-excess (black dashed-line), +10‰, is included as a reference [17].

Figure 3 shows a dual plot exhibiting *d*-excess variability (‰) of continental versus maritime TCs landfalls and passages within the Caribbean Sea and Atlantic Ocean basins, relative to the global *d*-excess mean of +10‰ (black dashed-line). These TCs produced a large spectrum of *d*-excess values below and above +10‰.

**Figure 4:**
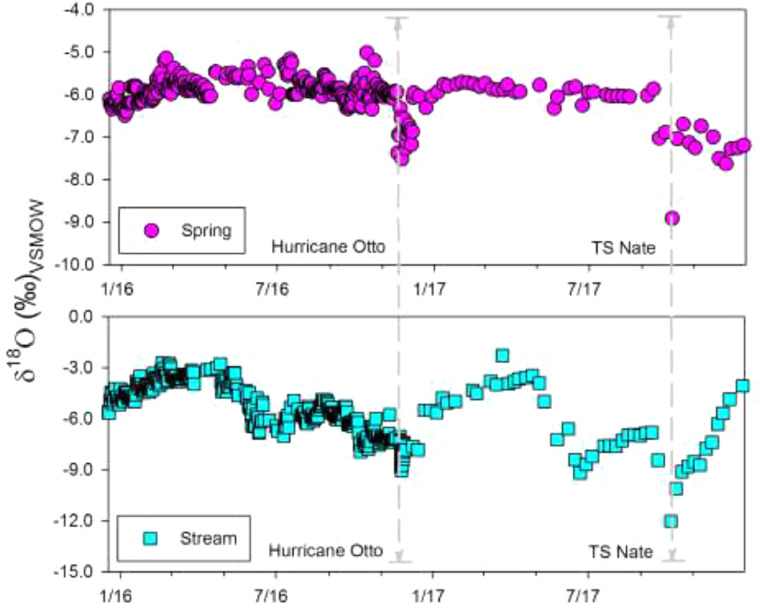
Daily and weekly δ^18^O (‰) time series (2015-2017) in spring and surface water comprising Hurricane Otto's landfall and Tropical Storm Nate's passage over and near Costa Rica, respectively. Upper panel: δ^18^O (‰) time series of a spring system in the Pacific slope of Costa Rica. Lower panel: δ^18^O (‰) time series of surface water in the Caribbean slope of central Costa Rica. The vertical grey-dashed lines denote Hurricane Otto's and Tropical Storm Nate's impact on the isotopic composition.

Figure 4 presents the propagation of isotopically distinct pulses from Hurricane Otto and Tropical Storm Nate in spring and surface water across the central mountain range of Costa Rica.

## 2 Experimental Design, Materials, and Methods

### 2.1 Sample collection

Rainfall samples (N=161) were collected using passive funnel devices [3] in central Costa Rica (Lat: 10.0945, Long: -84.0587, elevation: 1,949 m asl), Cuba (Cienfuegos) (Lat: 22.0657, Long: -80.4832, elevation: 35 m asl), and The Bahamas (Nassau) (Lat: 25.0739, Long: -77.4138, elevation: 11 m asl) with discrete time intervals (i.e., event-based, 6-hourly and daily) prior to, during, and after TCs continental and maritime landfalls or passages. Sampling efforts were designed to capture the first high frequency isotope composition of extreme TCs across the Caribbean Sea and Atlantic Ocean basins.

Spring (Pacific slope of central Costa Rica) (N=338) and surface water (Caribbean slope of central Costa Rica) (N=334) samples were collected on a daily/weekly (regular sampling frequency) and a 6-hourly basis during the direct and indirect influence of Hurricane Otto and Tropical Storm Nate in Costa Rica, respectively. Grab samples were collected on a weekly basis, whereas daily or 6-hourly samples were collected using an automated peristaltic sampler AS950 (Hach Company, Colorado, USA). All samples were transferred and stored in airtight 30 mL HDPE bottles and stored at 5°C until analysis.

### 2.2 Stable Isotopes analysis

Following all rainfall events, samples were immediately transferred and stored in airtight 30 mL HDPE bottles at 5°C until analysis. Hurricane Otto's samples were analyzed at the Stable Isotope Laboratory of the Northern Rivers Institute at the University of Aberdeen (Aberdeen, Scotland) using a DLT-100 laser analyzer (Los Gatos Research, Inc., California, USA) with a precision of ±0.6‰ for δ^2^H and ±0.1‰ for δ^18^O (1 σ). Hurricane Irma, Maria, and Dorian samples were analyzed at the Stable Isotopes Research Group laboratory at the Universidad Nacional (Heredia, Costa Rica) using a IWA-45EP water analyzer (Los Gatos Research, Inc., California, USA) with a precision of ±0.5‰ for δ^2^H and ±0.1‰ for δ^18^O (1 σ). Spring and surface water samples were analyzed at the Stable Isotopes Research Group laboratory at the Universidad Nacional (Heredia, Costa Rica) using a IWA-45EP water analyzer (Los Gatos Research, Inc., California, USA) and a L2120-i water analyzer (Picarro Inc., California, USA) with a precision of ±0.5‰ for δ^2^H and ±0.1‰ for δ^18^O (1 σ) in both instruments.

Stable isotope compositions are expressed as δ^18^O or δ^2^H = (R_s_/R_std_ - 1)•1000, where R is the ^18^O/^16^O or ^2^H/^1^H ratio in a sample (s) or standard (std) and reported in the delta-notation (‰) relative to V-SMOW/SLAP scale. The instruments’ accuracies were assessed with a combination of in-house and external water standards (SMOW and SLAP). Deuterium excess was calculated as *d*-excess = δ^2^H - 8•δ^18^O [18].

## Declaration of Competing Interests

The authors declare that they have no known competing financial interests or personal relationships that have, or could be perceived to have, influenced the work reported in this article.
